# Long-term outcome of allogeneic cultivated limbal epithelial transplantation for symblepharon caused by severe ocular burns

**DOI:** 10.1186/s12886-017-0403-9

**Published:** 2017-01-31

**Authors:** Jun Cheng, Hualei Zhai, Junyi Wang, Haoyun Duan, Qingjun Zhou

**Affiliations:** 1 Shandong Eye Institute, Shandong Academy of Medical Sciences, Qingdao Eye Hospital, 5 Yanerdao Road, Qingdao, 266071 China; 2 State Key Laboratory Cultivation Base, Shandong Provincial Key Laboratory of Ophthalmology, Shandong Eye Institute, Shandong Academy of Medical Sciences, 5 Yanerdao Road, 266071 Qingdao, China

**Keywords:** Cultivated limbal epithelial transplantation, Symblepharon, Thermal burn, Chemical burn

## Abstract

**Background:**

The therapeutic effects of allogeneic cultivated limbal epithelial transplantation (CLET) for symblephara at different degrees caused by ocular burns were evaluated in this study.

**Methods:**

A series of interventional cases were involved in this retrospective study. Eighty eyes (80 patients) with symblephara underwent CLET and the success rates of surgical treatment as well as corneal conditions and risk factors for recurrent symblepharon were analyzed.

**Results:**

The average age of patients was 32.4 ± 13.7 years (ranged from 4 to 60 years). The average follow-up time was 26.4 ± 13.6 months (ranged from 12 to 60 months). Symblepharon cases were caused by chemical burns (36 eyes) or thermal burns (44 eyes). The first surgical intervention achieved complete success in 40 eyes (50%), partial success in 25 eyes (31.3%), and failure in 15 eyes (18.8%). The rate of complete success was 85.0% in eyes with grade I/II symblephara, 51.5% in eyes with grade III eyes and 22.2% in eyes grade IV symblephara (*P* = 0.001). The treatment was completely successful in 23.1% of eyes with moderate or severe preoperative inflammatory action and 63.0% of eyes with mild or no inflammation (*P* = 0.000). The corneal conditions were improved in 43 eyes (53.8%), of which 21 eyes had improved visual acuity. The recurrence of symblepharon after the first CLET was positively correlated with symblepharon length (*P* = 0.003), preoperative inflammatory activity (*P* = 0.016) as well as postoperative cicatricial entropion and trichiasis (*P* = 0.038).

**Conclusions:**

CLET was effective on the recovery of anatomically deep fornixes in eyes caused by symblephara and corneal surface condition could be improved simultaneously. The success of surgical treatment was dependent on the effective control of inflammation and timely management of eyelid abnormalities.

## Background

The ocular chemical and thermal injuries which lead to symblepharon, corneal neovascularization, corneal scar and even loss of vision are not rare in young adults. Severe symblepharon after ocular burns is a challenging problem since it can result in tear reduction, tear film instability, cicatricial entropion and eye movement limitation [1, 2]. Symblephara due to chemical or thermal burns in most eyes are in combination with serious ocular surface dysfunction, including abnormal corneal surface, chronic inflammation, conjunctivalization, corneal vascularization, and poor epithelial integrity [3]. These conditions may lead to poor vision and abnormal appearance of patients. If we want to reconstruct the ocular surface of these patients, symblephara need to be managed first.

Symblephara can be treated by various approaches. When symblepharon is separated, the bulbar and/or palpebral conjunctival defects are exposed. To prevent re-adhesion, autologous conjunctival graft [2], oral mucosa [4, 5], amniotic membrane [6, 7], and nasal mucosa [8, 9] have been used to cover the defects. However, the amount of these tissues is usually not ample for severe cases. Allogeneic cultivated limbal epithelial transplantation (CLET) seems to have a favorable effect on total limbal stem cell deficiency (LSCD) [10–12]. Amniotic membrane carrying limbal stem cells is a good material for covering bare sclera, corneal stroma and tarsal plate. It can provide proliferated epithelial cells and the size can be chosen freely. After symblepharon is solved, the corneal condition is also improved, so that additional surgery can be performed later to restore the vision. The occurrence of immune rejection, which can facilitate cell death and failure of ocular surface reconstruction, is a problem of this technique. However, since most severe ocular surface disease is bilateral, surgeons have to use allograft donor cells. Herein, the long-term clinical outcomes and potential influencing factors in 80 patients treated by CLET for symblephara caused by chemical or thermal burns were evaluated in this study.

## Methods

### Study design

This retrospective study was approved by the institutional review board of Shandong Eye Institute and adhered to the tenets of the Declaration of Helsinki. The inclusion criteria were: (1) patients who had symblephara caused by chemical or thermal burns from January 2010 to September 2014; (2) CLET was performed for ocular surface reconstruction; (3) follow-up period was at least 12 months. Exclusion criteria included: (1) patients with severe dry eye (Schirmer test < 5 mm); (2) patients with less than 12 months follow-up; (3) patients who underwent CLET for causes other than chemical or thermal burns. Informed consents were obtained from all patients before surgery.

The severity of symblepharon was evaluated and graded I to IV based on photographic documentation in accordance with a previously reported grading system [13]. As for symblepharon lengths, grade I to IV was scored as 1 to 4, respectively. As for widths, grade a was scored as 1, grade b was scored as 2, and grade c was scored as 3. Absent inflammatory activity was scored as 0, mild inflammatory activity was scored as 1, moderate inflammatory activity was scored as 2, and severe inflammatory activity was scored as 3. An overall score was generated from 0 to 10 by adding the score of each item and a score of 10 indicated that the eyes were most severely affected. Meanwhile, the number of quadrants losing normal corneal limbal morphology was recorded.

### Cultivation of limbal epithelium

Fresh donor corneas were taken from Shandong Eye Institute Eye Bank. All donors were negative for syphilis, hepatitis B and C, and human immunodeficiency viruses 1 and 2. The circle area with a diameter of 8 mm in central cornea was used for penetrating keratoplasty, while the remaining corneal ring was used for culturing limbal epithelial cells. The corneal ring was digested in 1.2u/ml DispaseIIenzyme at 4 °C overnight and taken out to separate the limbal epithelial cell layer under a microscope the next day. The cell layer were digested into single cells by 0.25% trypsin and EDTA solution for 15 min. Human amniotic membrane (hAM) prepared and preserved by eye bank was used as a carrier. A 3 – 4 cm hAM sheet was de-epithelialised using 0.25% trypsin and EDTA solution for 15 min. Single limbal epithelial cells were seeded on hAM with a density of 1 ~ 2 × 10^5^. The limbal epithelial cells and hAM were then transferred into a cell culture plate pre-seeded with mitomycin C-inactivated NIH 3 T3 feeder cells, with medium changed every 2 days and 3 T3 feeder cells changed every 7 days. The medium used for culturing cells was prepared according to previous literatures [14, 15]. The cell sheets with 3 to 5 layers of stratification on the amniotic membrane were applied for clinical transplantation [14–17].

### Surgical techniques

According to the severity of symblepharon and the patient age, general anesthesia or peribulbar block anesthesia was adopted during the CLET surgery. The adhesions of conjunctiva and/or lid margin to the eye globe were carefully separated, and the bare sclera was exposed. The subconjunctival fibrovascular tissues were removed with minimal damage to conjunctival tissue. The superior rectus and inferior rectus were hooked before the scarred tissues around the rectus muscles were excised to ensure normal eye movement. Then the fibrovascular pannus on the surface of the cornea was cut to make the corneal surface as smooth as possible. After the bleeding vessels were electrocoagulated, the allogeneic limbal epithelial cell sheet cultured on human amniotic membrane was placed on the bare corneal stroma and sclera with the epithelial side oriented upward, and 10–0 nylon sutures were used to fix one edge of the amniotic membrance to the denuded parts of the corneal storma or to the limbus. The fixed position of another edge was set according to the symblepharon severity. In grage I/II symblepharon, the fixation was at the residual conjunctival edge. In grade III symblepharon, the amniotic membrane was fixed deep into the fornix and folded back to the residual palpebral conjunctival edge. In grade IV symblepharon, the fixation was made to the lid margin. For the symblephara involving both fornices, two pieces of such cultivated limbal epithelial cell sheets were put on the upper and lower bare sclera and palpebral conjunctiva, respectively, to create anatomically deep fornices. A bandage contact lens was placed at the end of the surgery, and tobramycin and dexamethasone eye ointments were administered.

### Postoperative therapy

During the first 3–5 days after surgery, intravenous hydrocortisone (2 mg/kg) was given per day, after which oral prednisolone (1 mg/kg) was used daily and tapered over a period of 2 to 3 months. During the first postoperative week, 20% autologous serum eyedrops were given topically once per 2 h, and then replaced with 0.02% fluorometholone eyedrops 4 times per day and cyclosporine A 1% eyedrops 4 times per day from the second week. Tobramycin and dexamethasone eye ointments were given every night. The patients were observed daily for the first postoperative week, weekly for the next 2 months, and monthly thereafter.

### Primary outcome measurement

The primary outcome measurement was the surgical effect evaluated by three levels including complete success, partial success, and failure. When an anatomically deep fornix was obtained, and no scar or motility restriction was found, complete success was achieved. Partial success was achieved when the score of recurrent symblepharon was more than 4 points lower than the preoperative score. If the recurrence score was less than 4 points lower than the original score, the treatment was regarded as failure. For eyes with more than one symblepharon, the symblepharon data with the highest grading were used. When the grade of each symblepharon was same, the analysis was based on the one with the comparatively worse outcome.

### Secondary outcome measurements

The secondary outcome measurements were: 1) risk factors for recurrence of symblepharon (including partial success and failure); 2) secondary surgery for recurrence; 3) intraoperative and postoperative complications; and 4) the BCVA of recipient eyes at the last follow-up or before the second surgery compared to baseline (before surgery).

### Statistical analysis

Statistical analysis was carried out with SPSS software version 19.0 (SPSS, Inc., Chicago, IL). The scores of symblephara before and after surgery between chemical and thermal burns were compared with independent-samples *t* test. The success rates for different factors were compared using chi-square analysis. Kaplan-Meier survival analysis was used to evaluate recurrence of symblepharon. Multivariate regression analysis was performed to verify whether the complete success after the first surgery was correlated with the factors of age, gender, cause of symblepharon, grade of symblepharon, preoperative inflammation, postoperative cicatricial entropion and trichiasis, duration of injury and surgery, loss of normal corneal limbal morphology, persistent epithelial defects, immune rejection, and incomplete eyelid closure. A P value of less than 0.05 was considered to be statistically significant.

## Results

### Patient characteristics

Eighty patients including 73 males and 7 females were recruited in this study. The average age of the patients was 32.4 ± 13.7 years (ranged from 4 to 60 years). The mean follow-up time was 26.4 ± 13.6 months (ranged from 12 to 60 months). The symblephara were caused by chemical burns in 36 eyes (acid burn, *n* = 11; alkali burn, *n* = 25) and thermal burns in 44 eyes (metal solution burn, *n* = 31; firework burn, *n* = 13). The injuries were bilateral in 12 patients and unilateral in 68 patients. The duration between injury and surgery was 3.5 months to 29 years (with a mean value of 21.3 ± 50.6 months). Nineteen eyes received allogeneic CLET within 6 months.

The mean score of symblepharon related to chemical burns was 6.3 ± 1.5, not significantly different from that of thermal burns (6.5 ± 1.3, P = 0.541). The length of the symblepharon was graded as I in 4 eyes, II in 16 eyes, III in 33 eyes, and IV in 27 eyes. The width of the symblepharon was graded as a in 20 eyes, b in 26 eyes, and c in 34 eyes. Before surgery, conjunctival inflammation was absent in 5 eyes, mild in 56 eyes, moderate in 16 eyes, and severe in 3 eyes. Fifty-seven eyes had total loss of normal corneal limbal morphology, and other eyes suffered partial LSCD.

### Primary outcomes

Forty eyes (50%) achieved complete success after the first surgery for symblepharon lysis, 25 eyes (31.3%) achieved partial success, and 15 eyes (18.8%) achieved failure. The outcome was closely related to the preoperative symblepharon severity and inflammatory activity. The treatment was completely successful in 17 (85.0%) of 20 eyes with grade I/II symblephara, 17 (51.5%) of 33 grade III eyes, and 6 (22.2%) of 27 grade IV eyes, while the rate of partial success was 10.0%, 30.3%, and 48.1%, respectively (*P* = 0.001). The complete success was achieved in 60.0%, 55.6%, and 39.4% of eyes graded a, b, and c, respectively (*P* = 0.019). The rate of complete success in eyes with moderate and severe preoperative inflammatory activity (23.1%) was less than that in eyes with mild or no inflammation (63.0%, *P* < 0.001). The cause of symblepharon also affected the surgical outcome, with the complete and partial success in 31 (70.5%) of 44 thermally injured eyes and in 34 (94.4%) of 36 chemically injured eyes (*P* = 0.020) (Table 1). The mean score of symblepharon induced by chemical burns at the last follow-up or before the second surgery was 1.6 ± 2.1, lower than that of symblepharon induced by thermal burns (2.6 ± 2.4, *P* = 0.045).

**Table 1:** Outcomes of fornix reconstruction

	No. of eyes	Complete success n (%)	Partial success n (%)	Failure n (%)	*P* value
Total	80	40 (50.0)	25 (31.3)	15 (18.8)	
Cause					
Thermal	44	18 (40.9)	13 (29.5)	13 (29.5)	0.020
Chemical	36	22 (61.1)	12 (33.3)	2 (5.6)	
Grade of symblepharon length					
Grade I/II	20	17 (85.0)	2 (10.0)	1 (5.0)	0.001
Grade III	33	17 (51.5)	10 (30.3)	6 (18.2)	
Grade IV	27	6 (22.2)	13 (48.1)	8 (29.6)	
Grade of symblepharon width					
Grade a	20	12 (60.0)	1 (5.0)	7 (35.0)	0.019
Grade b	27	15 (55.6)	9 (33.3)	3 (11.1)	
Grade c	33	13 (39.4)	15 (45.5)	5 (15.2)	
Preoperative inflammation					
≤1+	54	34 (63.0)	16 (29.6)	4 (7.4)	<0.001
≥2+	26	6 (23.1)	9 (34.6)	11 (42.3)	
Loss of normalcorneal limbal morphology					
≤3 quadrants	23	12 (52.2)	4 (17.4)	7 (30.4)	0.113
4 quadrants	57	28 (49.1)	21 (36.8)	8 (19.0)	
Time for surgery					
≤6 months	19	7 (36.8)	8 (42.1)	4 (21.1)	0.392
>6 months	61	33 (54.1)	17 (27.9)	11 (18.0)	

### Secondary outcomes

#### Complications

Fifteen eyes (18.8%) developed persistent epithelial defects, which were healed by wearing bandage contact lens (11 eyes) or permanent tarsorrhaphy (4 eyes). Seven eyes (8.8%) developed immune rejection at 1 to 3 months postoperatively, which was controlled by anti-rejection treatment as we previously reported [15]. Thirty-three eyes (41.3%) developed varying degrees of cicatricial entropion and (or) trichiasis, of which 13 eyes were managed by entropion correction, and other eyes were managed by a trichiasis electrolyzer. Twenty eyes (25.0%) had the problem of incomplete eyelid closure, of which 4 severe cases were managed by permanent tarsorrhaphy and eyelid reconstruction, and other mild cases were managed by wearing bandage contact lens until the ocular surface was stable.

### Visual acuity

**Fig. 1:**
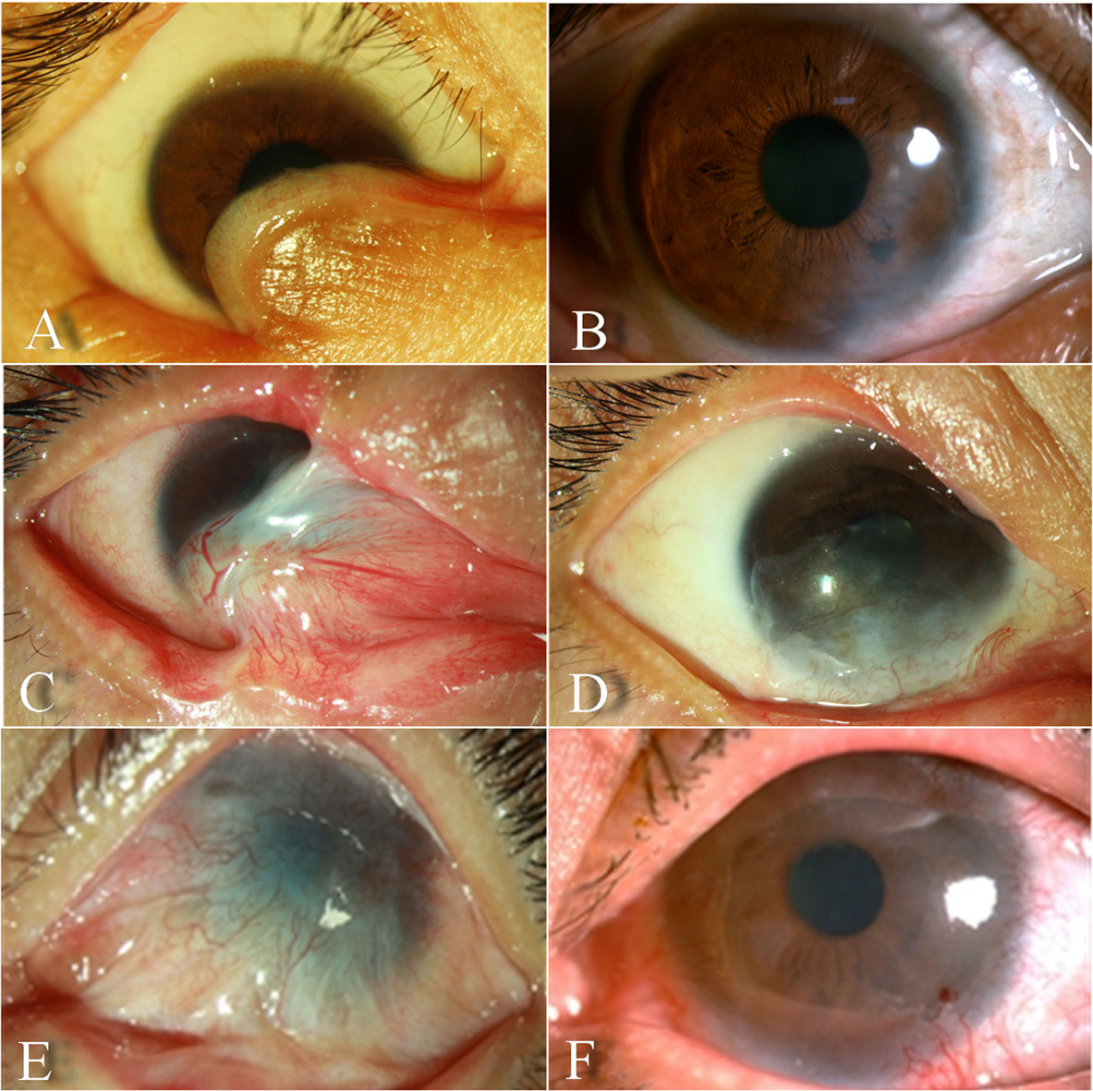
Grade IVb 0 symblepharon in the inferionasal fornix resulting from thermal burn (**a**); during the follow-up of 4.5 years, a deep fornix with complete success and a non-neovascularized cornea was noted (**b**). Grade IIIb 1+ symblepharon in the inferionasal and superonasal fornices resulting from thermal burn (**c**); a deep fornix without inflammation was noted together with a non-neovascularized cornea during 2 years of follow-up (**d**). Grade IIIb 1+ symblepharon in the superior fornix resulting from chemical burn (**e**); a deep fornix with complete success and a clear cornea was noted during 18 months of follow-up (**f**)

**Fig. 2:**
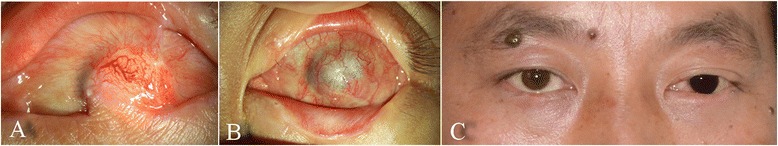
Grade IVa 1+ symblepharon in the inferior fornix resulting from thermal burn (**a**). a deep fornix was achieved 2 years later (**b**), and the appearance was improved through wearing a cosmetic contact lens (**c**)

Moreover, visual acuity (VA) was improved in 21 eyes (26.3%) and remained unchanged in 59 eyes (73.7%) before any additional surgery for visual restoration. The VA of two eyes were improved from hand movements to 20/400, three eyes improved by one Snellen lines, five eyes improved by two Snellen lines, two eyes improved by three Snellen lines, and nine eyes improved by five Snellen lines. Corneal conditions were improved in 43 eyes (53.8%) with prior conjunctivalization, of which 21 eyes had improved VA, and 22 eyes achieved the condition of additional lamellar or penetrating keratoplasty to improve VA (Fig. 1). Twenty of 23 eyes (87.0%) with fewer than three quadrants of losing normal corneal limbal morphology had alleviated corneal conjunctivalization, compared with 23 of 57 (40.4%) eyes with four quadrants of lossing normal corneal limbal morphology (*P* < 0.001). At the final follow-up, the appearance 29 eyes was improved through wearing cosmetic contact lenses (Fig. 2).

### Risk Factors for recurrence of symblepharon

**Fig. 3:**
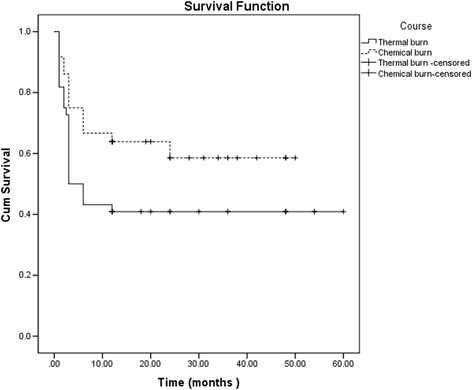
Survival analysis showing the clinical outcome of cultivated limbal epithelial transplantation for eyes with thermal burns and chemical burns

**Fig. 4:**
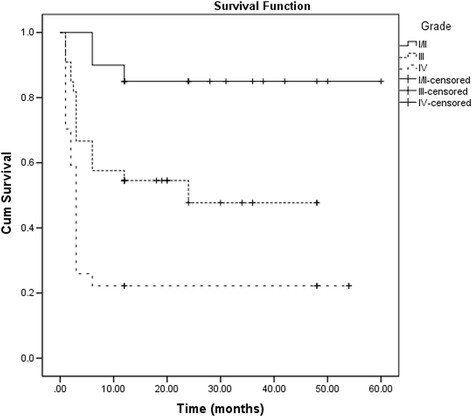
Survival analysis showing the clinical outcome of cultivated limbal epithelial transplantation for different grades of symblepharon

Symblephara recurred in 40 eyes with partial success or failure. Thirty-one eyes (77.5%) had recurrence within 3 months after surgery. The thermally burned eyes had a more rapid recurrence than the chemically burned eyes (*P* = 0.035, Fig. 3). The more severe for the length of the symblepharon, the more rapid the recurrence developed (*P* < 0.001, Fig. 4).

It was shown by multivariate regression analysis that the recurrence of symblepharon after the initial surgical intervention was positively correlated with the symblepharon length (*P* = 0.003), preoperative inflammatory activity (*P* = 0.016) as well as postoperative cicatricial entropion and trichiasis (*P* = 0.038). The postoperative cicatricial entropion and trichiasis was more common in thermal burns (63.6%, *n* = 21) than chemical burns (36.4%, *n* = 12). However, there was no significant correlation between the recurrence of symblepharon and gender, age, pathogeny, duration between injury and surgery, width of symblepharon, range of normal corneal limbal morphology loss, persistent epithelial defects (PED), immune rejection, or incomplete eyelid closure (Table 2).

**Table 2:** Risk factors for recurrence of symblepharon

	No. eyes	B value	SE	*P* value	RR (95% CI)
Gender					
Male	73	−0.574	0.687	0.403	0.563 (0.146–2.166)
Female	8				
Age					
≤18	12	−0.323	0.528	0.541	0.724 (0.257–2.039)
>18	68				
Cause					
Thermal	44	−0.514	0.456	0.260	0.598 (0.245–1.462)
Chemical	36				
Time for surgery					
≤6 months	19	−0.098	0.371	0.792	0.907 (0.438–1.875)
>6 months	61				
Grade of symblepharon length					
Grade I/II	20	0.917	0.311	0.003	2.503 (1.360–4.605)
Grade III	33				
Grade IV	27				
Grade of symblepharon width					
Grade a	20	0.195	0.301	0.517	1.215 (0.674–2.190)
Grade b	27				
Grade c	33				
Preoperative inflammation					
≤1+	54	1.003	0.415	0.016	2.726 (1.209–6.147)
≥2+	26				
Loss of normal coneal limbal morphology					
≤3 quadrants	23	−0.014	0.458	0.976	0.986 (0.402–2.423)
4 quadrants	57				
Postoperative PED					
Yes	15	0.444	0.425	0.297	1.558 (0.677–3.588)
No	65				
Immune rejection					
Yes	7	0.661	0.839	0.431	1.936 (0.374–10.025)
No	73				
Cicatricial entropin and (or) trichiasis					
Yes	33	0.774	0.373	0.038	2.168 (1.044–4.504)
No	47				
Incomplete eyelid closure					
Yes	20	0.637	0.387	0.100	1.891 (0.885–4.040)
No	60				

### Secondary surgery

Of 40 eyes with recurrence of symblephara after the first attempt, 27 eyes underwent a second surgery, resulting in complete success in 21 eyes (77.8%) and partial success in 2 eyes (7.4%). The second surgical management included allogeneic CLET in 17 eyes, oral mucosa transplantation in 6 eyes, and autologous cultivated oral mucosal epithelial transplantation in 4 eyes, resulting in complete success in 12 eyes (70.6%), 6 eyes (100%), and 3 eyes (75.0%), respectively (Table 3).

**Table 3:** Secondary surgical procedure and outcomes

First surgical result	Total	Secondary surgery	Surgical procedure	No. eyes	Complete success (n)
Partial success	25	17	Allogeneic CLET	10	8
			Oral mucosa transplantation	3	3
			Autologous cultivated oral mucosal epithelial transplantation	4	3
Failure	15	10	Allogeneic CLET	7	4
			Oral mucosa transplantation	3	3

### Outcomes for pediatric cases

There were 10 (12.5%) pediatric cases in this group, with an average age of 9.1 ± 3.5 years and average follow up time of 23.2 ± 12.0 months. The symblephara were caused by alkali burns in 2 eyes, and fireworks burns in 8 eyes. All injuries were unilateral. The length of the symblepharon was graded as II in 1 eye, III in 6 eyes, and IV in 3 eyes. The width of the symblepharon was graded as a in 4 eyes, b in 5 eyes, and c in 1 eye. Before surgery, conjunctival inflammation was mild in 9 eyes, moderate in 1 eye. Three eyes achieved complete success after the first surgery for symblepharon lysis, 3 eyes achieved partial success, and 4 eyes achieved failure. One eye developed persistent epithelial defects, which were healed by wearing bandage contact lens. One eye developed immune rejection at 1 month postoperatively, which was controlled by anti-rejection treatment. Eight eyes developed cicatricial entropion and (or) trichiasis, of which 4 eyes were managed by entropion correction. Three eyes had mild incomplete eyelid closure, which were untreated since the ocular surface was not affected. Of 7 eyes with recurrence of symblephara after the first attempt, 3 eyes underwent a second surgery, including allogeneic CLET in 2 eyes resulting in complete success, and autologous cultivated oral mucosal epithelial transplantation in 1 eye resulting in failure. Visual acuity of the 10 pediatric cases which were stable in hand movements or counting figure had no improvement.

## Discussion

Chemical and thermal injury could lead to severe ocular surface disorders, such as corneal neovascularization, corneal epithelial defects, corneal ulcer formation, corneal scars, and symblepharon formation, which affect the patient's visual function and appearance seriously. Although CLET is effective in treating LSCD resulted from ocular surface burns [18–20], the ocular surface reconstruction process for severe chemical and thermal burns with symblepharon is complicated. Sotozono et al [21] considered that symblepharon and eyelid disorders were important factors for a successful result of cultivated oral mucosal epithelial sheet transplantation. Prabhasawat et al [22] reported the failure of CLET in severe chemical injury with symblepharon and eyelid disorder. Thus, release of symblepharon and correction of eyelid abnormality are the first step of the management.

Prabhasawat et al [22] reported that CLET could decrease conjunctival inflammation and eliminate symblepharon as well as improve corneal surface conditions. There has been no specific reports about the effect of CLET on releasing symblepharon. In the present study, CLET was confirmed as an effective method for symblepharon caused by severe chemical and thermal burns. The success (complete and partial) rate of the first surgical attempt of fornix reconstruction was 81.3%, and the complete success rate was 50%. Kheirkhah et al [13] reported the success (complete and partial) rate of 85.2% and complete success rate of 63.9% after cicatrix lysis and amniotic membrane transplantation with autologous oral mucosal graft or conjunctival graft. As an ideal covering on bare sclera, corneal stroma, tarsal plate and cultivated corneal epithelial sheets on the amniotic membrane could provide proliferative epithelial cells and prevent the invasion of fibrovascular tissue over the graft. It was also free to choose the sheet size.

In this study, the surgical success rate was different in eyes affected by chemical and thermal burns. Patients whose symblephara was caused by thermal burns had a worse prognosis when the symblepharon severity was similar. The recurrence of symblephara also occurred earlier in eyes with thermal burns than chemical burns. Thermal burns more commonly led to severe eyelid margin and tarsal defection than chemical burns. After symblepharon lysis, the upper or lower eyelid cicatricial entropion with various degrees of tarsal atrophy was sometimes found. Eyelid condition is an important factor for maintaining ocular surface stability and avoiding complications such as infection and persistent epithelial defects [21]. It was noted that the recurrence of symblepharon was related to postoperative cicatricial entropion and trichiasis, but not related to the cause of symblepharon. Prabhasawat et al [22] also reported that severe corneal stromal abnormalities and severe eyelid deformity after CLET, complicated by postoperative epithelial defection and infection, resulted in treatment failures. It is indicated that the eyelid function plays a key role in the surgical success. In cases combined with an eyelid abnormality, eyelid surgery should be performed to correct entropion, trichiasis, or lagophthalmos.

Surgical failure may more easily happen in symblephara at higher grades [6, 13, 23]. The length of symblepharon seemed to be a more significant factor than the width of symblepharon according to our multivariate regression analysis result. Grade III and IV symblepharon meant very few conjunctiva residues, which needed some tissues similar to conjunctiva to cover the defection [24–26]. The effect of cultivated limbal epithelium for severe symblephara in this study (total success rate in grade III and IV was 81.8% and 70.3%, respectively) was similar to that of oral mucosa graft. [13, 27, 28]

Kheirkhah et al [13] reported that the surgical results were not linked closely with the inflammation degree in the symblepharon, but there was correlation between persistent postoperative inflammation and pterygium recurrence [29]. Sejpal et al [19] considered that postoperative inflammation may play a significant role in the outcome of CLET. In their series, eyes undergoing CLET within 4 months after the injury had a higher failure rate, indicating that low-grade smoldering inflammation might persist for a prolonged period of time, especially in cases with acute alkali injury. We agreed with this view, and most of patients in our research underwent the surgery 6 months after injury. But there are a small portion of patients who underwent surgery within 6 months. The results showed that the complete success rate in patients who underwent surgery within six months after injury was lower than that in patients who underwent surgery more than six months. But there was no difference in statistical analysis, which might be resulted from the large difference in case number between the two groups. This study is a retrospective study. The chosen surgical indication was the period when ocular surface of patients were in relative stable phase, but the specific time range from injury to surgery was not defined in detail. Therefore, some patients who were injured less than 6 months before surgery but exhibited relative ocular surface stability also underwent the surgery. However in the course of our research, it was gradually found that the time range of more than 6 months from injury to surgery was more suitable for the surgery. So we gradually strengthened the control on the timing of surgery in the follow-up study. This might also lead to no significant difference in statistical analysis.

In our series, the recurrent symblephara occurred more often in eyes with moderate and severe preoperative inflammation (RR = 2.726, *P* = 0.016). The total success rate was 92.6% in eyes with mild or no preoperative inflammation, but only 57.7% in eyes with moderate and severe inflammation. Moreover, the time of surgery did not affect the eventual outcome in our series, similar to the study of Sangwan et al. [30] Immune rejection was observed in seven eyes (8.8%) on postoperative week 1 to 3. Chronic inflammation due to immune rejection accelerated the disappearance of amniotic membrane, which might lead to the recurrence of symblepharon [31–36]. We preferred to use allogeneic transplantation out of the following considerations. First, although it was shown by most reports that obtaining limbal tissues from the health eye would not result in negative effect, it could still cause certain damage to the healthy eye more or less. Second, patients would have some concerns about the operation to the health eye. Third, more sufficient corneal limbal epithelial cells could be obtained from donor allogeneic cornea. Forth, while using allograft, the rejection rate could be kept lower (8.8%) by a small amount of anti-rejection drugs.

CLET could also improve the corneal surface condition simultaneously in eyes with symblephara. In this study, most of the eyes had symblephara at grade III and IV, with severe corneal conjunctivalization and corneal stromal scarring. At the final follow-up, 29 eyes obtained improved appearance through wearing cosmetic contact lenses, and 43 eyes had alleviated corneal conjunctivalization besides symblepharon lysis. Partial or total LSCD could impact on the corneal condition, but could not affect the success rate of symblepharon lysis [37]. Some eyes needed a repeated surgery. The primary surgery might partially improve the condition of symblepharon and corneal surface, while the second surgery could further resolve the residual symblephara. The success rates of the initial and repeated surgeries were similar. Two-thirds to four-fifths of the patients who did not achieve favorable outcomes after primary surgery could be successfully treated by a repeated procedure [30, 37, 38].

## Conclusions

Allogeneic CLET is effective in achieving an anatomically deep fornix in eyes with symblephara due to severe chemical and thermal injuries. Meanwhile, corneal surface condition could be improved, which makes it possible to perform a further keratoplasty and improve the survival chance of corneal grafts. However, the success rate of this surgery depends on the grade of symblepharon, preoperative inflammatory activity as well as postoperative cicatricial entropion and trichiasis. Therefore, effective control of inflammation before and after surgery, and timely management of eyelid abnormalities are required. If the primary surgery does not achieve a complete success, a repeated surgery could be considered.

## Abbreviations

CLET: Cultivated limbal epithelial transplantation
LSCD: Limbal stem cell deficiency
PED: Persistent epithelial defect
